# Integrative Multi-Omics Analysis Reveals Key Metabolic Regulators and Prognostic Biomarkers in Pediatric and Adult Thyroid Cancer

**DOI:** 10.7150/jca.117034

**Published:** 2025-07-28

**Authors:** Bin Ling, Mengran Tian, Jinmiao Wang, Wei Luo, Jialong Yu, Xing Wan, Qiman Dong, Ming Gao, Min Zhao, Xiangqian Zheng, Xianhui Ruan

**Affiliations:** 1 Center for Precision Cancer Medicine and Translation Research, Tianjin Cancer Hospital Airport Hospital, Tianjin, 300060, China.; 2 Department of Thyroid and Neck Tumor, Tianjin Medical University Cancer Institute and Hospital, National Clinical Research Center for Cancer, Tianjin's Clinical Research Center for Cancer, Key Laboratory of Cancer Prevention and Therapy, Tianjin 300060, China.; 3 Department of Thyroid and Breast Surgery, Tianjin Union Medical Center, The First Affiliated Hospital of Nankai University, Tianjin, 300121, China.; 4 School of Science, Technology and Engineering, University of the Sunshine Coast, Maroochydore DC, Queensland 4558, Australia.

**Keywords:** multi-omics integration, thyroid carcinoma (TC), proteomics, metabolomics, RNA sequencing (RNAseq), energy metabolism, immunotherapy

## Abstract

Thyroid cancer, including papillary thyroid carcinoma (PTC) and anaplastic thyroid carcinoma (ATC), exhibits distinct molecular characteristics in adult and pediatric populations. Understanding these differences is vital for identifying therapeutic targets and prognostic biomarkers. We performed an integrative multi-omics analysis combining proteomics, phosphoproteomics, metabolomics, and RNA sequencing data from adult and pediatric thyroid cancer cohorts. Differential expression analyses were conducted for all the multi-omics data with false discovery rate adjustments. Enzyme mapping of metabolites was performed using MetaBridge, while cross-omics integration revealed 46 key genes associated with reprogrammed energy metabolism. Clinical relevance was evaluated through survival analyses on cBioPortal and KM plotter platforms, and immunotherapy responses were assessed based on gene expression profiles. The 46 identified genes, primarily involved in mitochondrial energy metabolism and oxidative phosphorylation, were strongly associated with poor disease-free and overall survival in PTC and ATC patients. In ATC, a high tumor mutation burden correlated with worse outcomes, underscoring its prognostic value. Additionally, seven genes (AK2, SUCLG2, NDUFV2, GLUD1, HADHA, ALDH1A1, and NADSYN1) were linked to improved responses to anti-PD-1 immunotherapy, highlighting their potential as biomarkers for treatment stratification. Furthermore, functional studies reveal that AK2 plays a key role in thyroid cancer progression. This study offers critical insights into thyroid cancer biology and provides a foundation for targeted therapies and personalized immunotherapy strategies to improve patient outcomes.

## Introduction

Thyroid cancer is the most prevalent endocrine malignancy, encompassing a spectrum of histological subtypes 1, with papillary thyroid carcinoma (PTC) and anaplastic thyroid carcinoma (ATC) being the most common and aggressive forms, respectively. The diagnostic challenges of common biomarkers in pediatric vs. adult PTC arise from age-related differences in mutation prevalence, limitations in FNA cytology, and biomarker overlap with benign conditions 2, 3. A comprehensive molecular approach integrating genomic, transcriptomic, and proteomic data is essential for improving diagnostic accuracy and tailoring risk-based management strategies for both populations.

Proteomics and phosphoproteomics provide insights into protein expression and post-translational modifications, respectively, elucidating dynamic cellular processes 4, 5. Metabolomics offers a snapshot of metabolic alterations, essential for understanding cancer cell metabolism 6, while RNA sequencing (RNAseq) reveals transcriptional changes that drive oncogenic pathways 7. Despite the wealth of data, integrating multi-omics information to pinpoint critical genes and pathways remains a significant challenge 8. Moreover, the identification of clinically relevant biomarkers that can predict patient outcomes and responses to therapies, such as immunotherapy, is paramount for advancing personalized medicine in thyroid cancer treatment.

In this study, we employed an integrative multi-omics approach to analyze proteomic, phosphoproteomic, metabolomic, and transcriptomic data from both adult and pediatric thyroid cancer cohorts. By intersecting differentially expressed genes across these datasets, we identified a core set of 46 genes implicated in energy metabolism reprogramming. Further, we investigated the clinical relevance of these genes through survival analyses and assessed their potential as biomarkers for immunotherapy response using publicly available genomic and clinical datasets. The comprehensive integration of multi-omics data not only enhances our understanding of the molecular landscape of thyroid cancer but also identifies novel therapeutic targets and prognostic markers. This study aims to bridge the gap between molecular alterations and clinical outcomes, providing a foundation for the development of targeted and personalized treatment strategies in thyroid cancer.

## Materials and Methods

### Data Sources and Study Design

To comprehensively characterize the molecular landscape of papillary thyroid carcinoma (PTC), we utilized two independent multi-omics datasets 3, 5, integrating transcriptomic, metabolomic, proteomic, and phosphorylated (phospho)-proteomic data. The first dataset consisted of an integrated proteogenomic and metabolomic investigation of 102 Chinese PTC patients, which provided a detailed molecular profile of adult PTC 5. Proteomic profiling was performed on 37 paired tumor-normal tissue samples, quantifying 3,147 proteins using mass spectrometry. This dataset allowed for a comparative analysis of tumor-specific proteomic alterations in adult PTC.

To further examine the molecular differences between pediatric and adult PTC, we leveraged a publicly available pediatric PTC proteomics dataset 3, which included 83 pediatric benign (PB) and 85 pediatric malignant (PM) PTC samples. This dataset quantified 10,426 proteins using high-resolution mass spectrometry, enabling a broad assessment of proteomic changes in pediatric thyroid cancer. The comparison between pediatric and adult datasets aimed to uncover distinct molecular signatures and potential age-dependent variations in tumor biology.

### Proteomic Data Processing and Statistical Analysis

For the adult PTC dataset, differential expression analysis was conducted to compare protein abundance between tumor and adjacent normal tissues. The Wilcoxon signed-rank test (two-sided, paired) was applied to identify significantly dysregulated proteins. Expression differences were quantified using log2 fold changes (Log2FCs), and multiple hypothesis testing was controlled using the Benjamini-Hochberg method to adjust p-values and control the false discovery rate (FDR). This statistical approach ensured robust identification of differentially expressed proteins associated with tumor progression.

In the pediatric PTC dataset, protein expression differences between pediatric malignant (PM) and pediatric benign (PB) samples were assessed using the Wilcoxon rank-sum test (two-sided, unpaired). A total of 243 significantly dysregulated proteins were identified based on adjusted p-values and Log2FCs. Further pathway enrichment analysis highlighted the involvement of inflammatory and immune-related pathways, suggesting a potential role of immune dysregulation in pediatric thyroid cancer progression. These analyses provided insights into key molecular mechanisms driving PTC across different age groups and emphasized the distinct proteomic landscape of pediatric and adult tumors.

### Phospho-Proteomics Analysis of PTC Tumor and Normal Tissues

From the first dataset, which included 102 Chinese PTC patients 5, we obtained phospho-proteomics data comprising 652 phosphoproteins, profiled from 37 paired tumor-normal tissue samples. The phosphoproteomics data were analyzed using the Wilcoxon signed-rank test (two-sided, paired), following the same approach as the proteomics analysis. Differentially phosphorylated proteins were identified based on site-specific phosphorylation abundance. Log2 fold changes (Log2FCs) and adjusted p-values (Benjamini-Hochberg correction) were used to prioritize statistically significant phosphorylation sites.

### Metabolomics Data Processing and Enzyme Mapping

To utilize the available metabolomics data from the first dataset 5, we obtained metabolite profiling of 503 metabolites from 37 paired tumor and normal tissue samples. The data were processed to identify significantly differentially expressed metabolites between tumor and normal conditions. To map metabolites to their corresponding KEGG (Kyoto Encyclopedia of Genes and Genomes) enzymes, we used the MetaBridge tool 9. A total of 334 differentially expressed metabolites were successfully linked to relevant KEGG enzymes. In brief, MetaBridge performs automated mapping by linking each metabolite to its corresponding enzyme(s) based on the KEGG database annotations. This process involves several steps. Firstly, the list of differentially expressed metabolites, along with their KEGG compound identifiers, was uploaded to the MetaBridge platform. Then, MetaBridge cross-referenced the metabolite identifiers with the KEGG database to identify associated enzymes. This step ensures that each metabolite is accurately linked to its relevant biochemical pathways and enzymatic reactions. One metabolite could be mapped to multiple enzymes. Lastly, we collected a comprehensive list of KEGG enzymes corresponding to the differentially expressed metabolites.

### RNA-Seq Analysis and Differential Gene Expression Identification

The RNA-seq data from the first dataset 5 included raw read counts mapped to 16,925 genes, with expression levels normalized as FPKM (Fragments Per Kilobase of transcript per Million mapped reads). The dataset comprised 92 tumor tissue samples and 34 paired normal tissue samples, providing a comprehensive view of transcriptomic alterations in PTC. To identify differentially expressed genes (DEGs) between tumor and normal tissues, we utilized DESeq2 10. DEGs were filtered based on strict criteria, requiring an absolute log2 fold change (|log2FC| > 1) and a corrected P-value < 0.05, using the false discovery rate (FDR) method to control for multiple testing. These thresholds ensured that the identified DEGs reflected statistically significant and biologically meaningful changes in gene expression between conditions.

### Gene Set Analysis

The intersection of gene lists was visualized using the VennDiagramWeb tool 11, a web application that generates highly customizable Venn and Euler diagrams. Genes present in at least three out of the five input lists were included. The five lists comprised two proteomics differentially expressed results, one RNAseq differentially expressed gene list, one differential phosphorylation result, and enzymes mapped based on differential metabolites. In the set analysis, we extracted subsets that had at least two additional pieces of evidence from the input lists, in addition to the enzymes mapped based on differential metabolites. This visualization highlighted 46 intersecting genes for subsequent analysis.

### Cancer Hallmark Annotation

To conduct a cancer hallmark analysis using 46 genes of interest, we utilized a consensus gene set for cancer hallmarks to streamline data comparison and integration 12. In brief, a total of 1,574 core genes are associated with ten cancer hallmarks. By mapping our 46 genes to their most prominent hallmarks, we categorized them into relevant groups and generated enrichment scores for each hallmark, such as "tissue invasion and metastasis" and "sustained angiogenesis." The analysis produced statistical metrics, including p-values and enrichment scores, which highlighted potential drug targets linked to specific cancer hallmarks. This approach provided a comprehensive overview of the relationship between the selected genes and cancer hallmarks, aiding in the identification of potential biomarkers and therapeutic targets.

### Functional Enrichment Analysis

To examine the functional roles of the 46 genes identified at the intersection of multiple omics analyses, we performed comprehensive functional annotation and pathway analyses. The analysis pipeline integrated multiple approaches to systematically characterize the biological significance of these genes. In this study, functional annotation and enrichment analyses were performed using ToppFun 13, a web-based tool that is part of the ToppGene Suite. ToppFun provides comprehensive gene list enrichment analysis and includes various annotation categories such as GO terms, KEGG pathways, phenotypes, drugs, and diseases. The tool employs hypergeometric distribution with FDR correction for statistical analysis of enrichment results.

For pathway analysis, we leveraged the KEGG to map the genes to established biological pathways. This approach helped identify key molecular networks and signaling cascades associated with our gene set. In our functional enrichment analysis, we prioritized the Gene Ontology (GO) cellular component results from ToppFun, as the GO biological processes and molecular functions categories showed considerable redundancy with the KEGG pathway findings. This targeted approach allowed us to focus on the unique spatial organization of our genes while avoiding duplicate functional information. Statistical significance was assessed using Fisher's exact test, with p-values adjusted for multiple testing using the False Discovery Rate (FDR) method.

Results were visualized using bubble plots, where each bubble represents an enriched term. The size of each bubble indicates the number of genes associated with that term, while the color intensity reflects the statistical significance (FDR-adjusted p-value). This visualization method effectively communicates both the scale and significance of the enriched functional categories. This analysis framework provided a systematic approach to understand the biological context of the identified genes, revealing their potential roles in cellular components and pathways relevant to thyroid cancer development and progression.

### Cancer Clinical and Genetic Data Mapping

All the sample based mutational analysis were based on the cBioPortal 14, an online server for visualization, and analysis of multidimensional cancer genomics data. This comprehensive approach not only aids in elucidating the potential biological roles of interested genes but also provides a systems-level perspective on their contributions to cellular and molecular processes.

Kaplan-Meier survival curves were generated using cBioPortal for the PTC cohort. Patients were grouped into altered and unaltered groups based on mutations in the 46 genes. Log-rank tests were applied to determine statistical significance for disease-free survival (p < 0.05). Overall survival analysis was conducted for ATC samples using cBioPortal. The altered and unaltered groups were defined similarly to Figure 4A. Hazard ratios were calculated with 95% confidence intervals, and statistical significance was determined using logrank tests with p-values indicating statistical significance. Survival curves were generated with numbers at risk displayed below each graph.

Oncotreemap in cBioPortal was used to classify ATC samples based on the presence or absence of mutations in the 46 genes. This visualization highlighted the clinical relevance of these mutations in defining ATC subtypes. Tumor mutation burden (TMB) scores for ATC samples were retrieved from cBioPortal. TMB differences between the altered and unaltered groups were compared using the Wilcoxon rank-sum test. Results were visualized as box plots.

The KM plotter offers a robust platform for survival analysis in immunotherapy cohorts, including a dataset of 520 patients treated with anti-PD-1 therapy 15. In a word, KM plotter provides a comprehensive database containing both gene expression and clinical data. Therefore, it is efficient to screen multiple datasets with clinical response and transcriptomic data from various cancers, focusing on anti-PD-1 therapies like nivolumab and pembrolizumab. Researchers can input genes of interest into the platform's immunotherapy module to assess survival outcomes based on gene expression levels. The platform automatically divides patients into high and low expression groups using optimal cutoff values, generating Kaplan-Meier plots with associated hazard ratios (HR) and log-rank p-values.

To perform the analysis, we should navigate to the immunotherapy section of kmplot.com and select "Anti-PD-1 treatment." After entering the gene symbol(s) of interest, the platform generates survival curves illustrating the relationship between gene expression and treatment outcomes. Results include key statistical metrics such as hazard ratios with 95% confidence intervals, log-rank p-values, and the number of patients at risk at various time points. This analysis helps identify potential biomarkers for immunotherapy response and provides insights into the relationship between gene expression and treatment effectiveness in cancer patients receiving anti-PD-1 therapy.

### Cell Proliferation and Migration Ability

AK2 expression was silenced in CAL-62 and KTC-1 cells using AK2-specific small interfering RNA (siRNA) (Genema Gene), following the manufacturer's protocol with the supplied transfection reagent. The efficiency of gene knockdown was validated by quantitative real-time PCR (qRT-PCR). Primer sequences were as follows: Human AK2—Forward: 5'-TCCTACCACGAGGAGTTCAACC-3', Reverse: 5'-TGGTAGGCTTGCAGGCGGATTT-3'; Human Beta-actin (ACTB)—Forward: 5'-CACCATTGGCAATGAGCGGTTC-3', Reverse: 5'-AGGTCTTTGCGGATGTCCACGT-3'. For the CCK-8 assay, cells were trypsinized, counted, and plated into 96-well plates at 1,000 cells per well. Cell viability was measured every 24 hours by adding CCK-8 reagent and recording absorbance. For colony formation, cells were seeded in 6-well plates at 1,000 cells per well. Media were refreshed every 2-3 days. Once colonies formed, they were fixed, stained, and counted. In the Transwell migration assay, 5,000 cells in serum-free medium were seeded into the upper chamber, while the lower chamber contained serum-supplemented medium. After 24 hours, cells on the underside of the membrane were fixed, stained, and visualized microscopically.

## Results

### Integrative Multi-Omics Approach for Pediatric and Adult Thyroid Cancer Analysis

To comprehensively investigate thyroid cancer progression in both pediatric and adult cohorts, we employed an integrative multi-omics approach by combining proteomics, phosphoproteomics, metabolomics, and RNA sequencing (RNA-seq) data from two recently published datasets of Chinese PTC patients. The first dataset included an integrated transcriptomic, metabolomic, proteomic, and phospho-proteomic analysis of 102 adult PTC patients 5, while the second dataset focused exclusively on proteomic profiling, comprising 83 pediatric benign (PB) and 85 pediatric malignant (PM) PTC samples 3.

**Figure 1:**
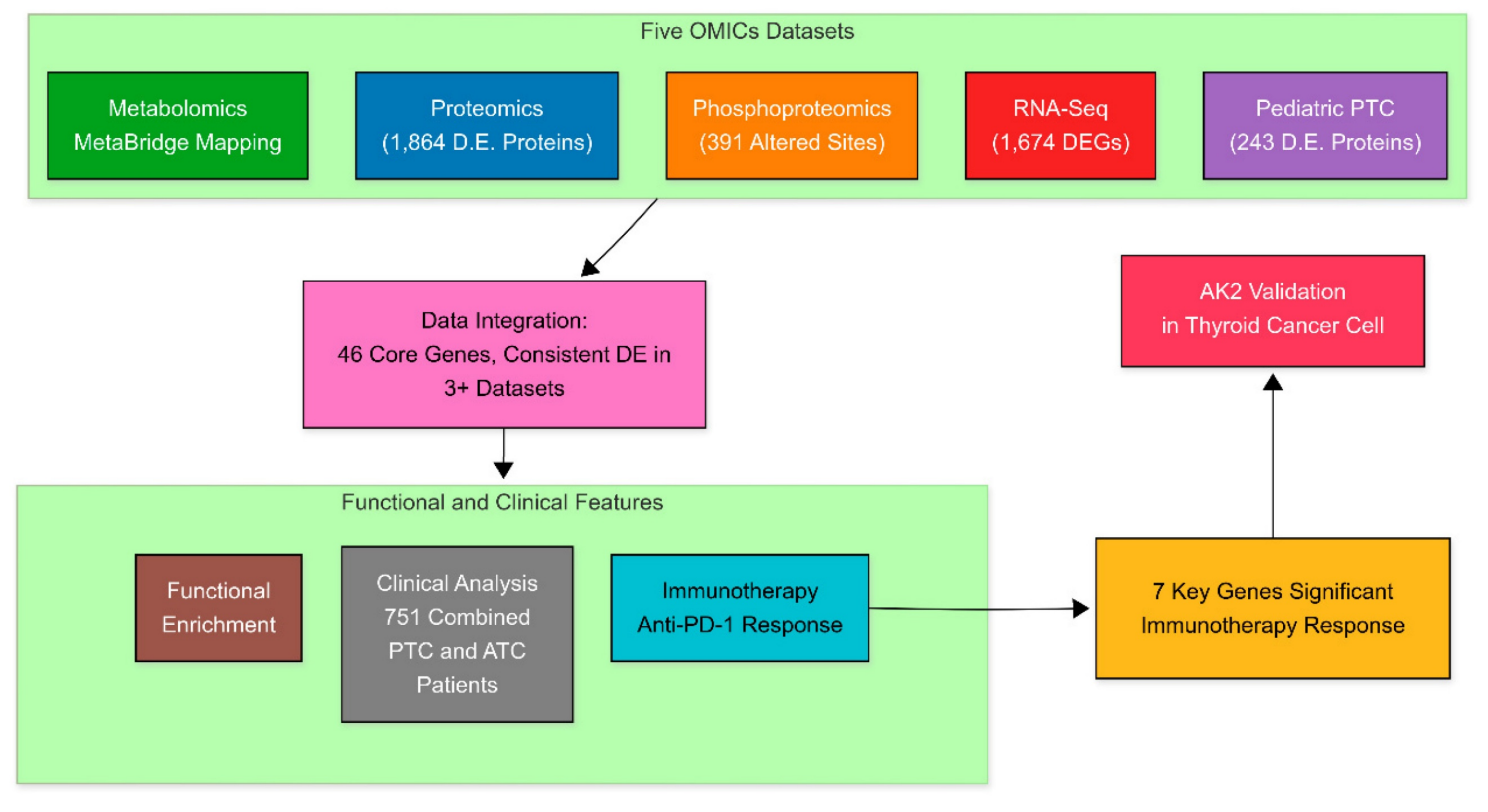
Multi-omics integration identifies therapeutic targets in thyroid cancer. This workflow combines proteomic, phosphoproteomic, transcriptomic, and metabolomic data from adult and pediatric papillary thyroid cancer (PTC) cohorts. Cross-omics analysis identified 46 core genes, which were clinically validated in 751 patients and functionally characterized through immunotherapy response profiling. Seven genes demonstrated significant immunotherapeutic relevance, with the top candidate AK2 experimentally confirmed in thyroid cancer cell lines.

As illustrated in Figure 1, the analytical workflow integrates five interconnected multi-omics components, represented by distinct modules. The proteomics analysis identified 1,864 differentially expressed proteins, phosphoproteomics revealed 391 altered phosphorylation sites, RNA sequencing detected 1,674 differentially expressed genes, and pediatric PTC proteomics highlighted 243 dysregulated proteins. Unlike other omics layers that could be mapped directly to gene symbols, metabolomics analysis resulted in metabolite-level outputs. To integrate these findings, MetaBridge was used to systematically map differentially expressed metabolites to their corresponding KEGG enzymes, enabling deeper insights into metabolic dysregulation and pathway alterations in PTC.

At the core of the workflow, a robust data integration module consolidates multi-omics datasets—including transcriptomics, proteomics, phosphoproteomics, and metabolomics—to identify 46 key genes associated with energy metabolism. To address confounding factors such as batch effects, technical variability, tumor purity, and cellular heterogeneity, each dataset underwent rigorous quality control, normalization, and independent differential expression analysis using statistical methods such as the Wilcoxon Signed-Rank Test and DESeq2, with adjustments for multiple testing to reduce bias and false positives. Enzyme mapping of metabolic metabolites was conducted using MetaBridge to standardize pathway-level interpretations and enhance cross-platform comparability. The integrated molecular signatures were then evaluated through clinical relevance assessments (mutation analysis of 751 thyroid cancer patients), immunotherapy response profiling (anti-PD-1 treatment outcomes), and functional enrichment analyses (cancer hallmarks, KEGG pathways, and GO cellular components). This comprehensive, stepwise approach offers a biologically meaningful and scalable framework for multi-omics analysis in cancer research.

### A Multi-Omics Perspective on Molecular Alterations Across Age Groups

**Figure 2:**
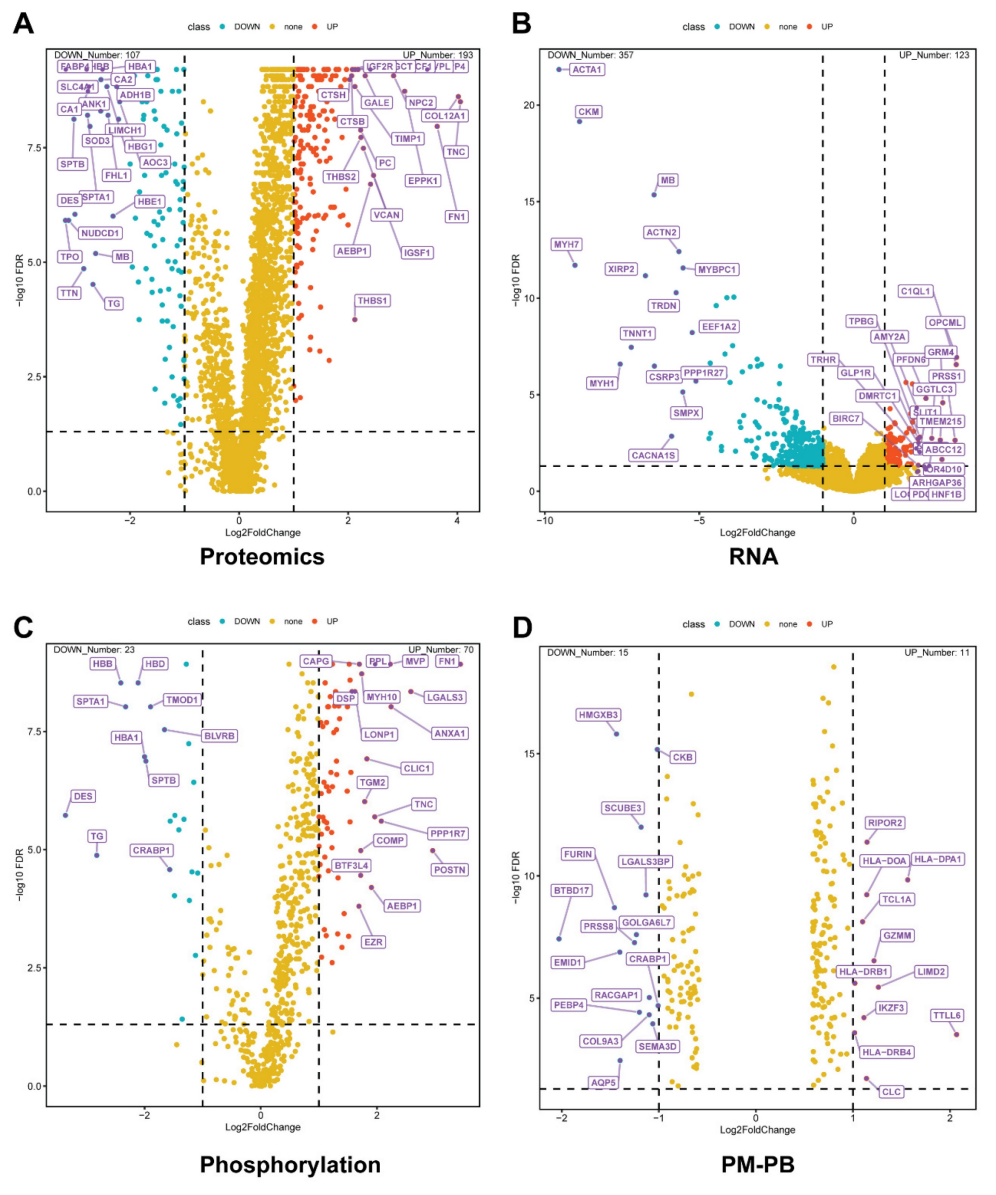
Multi-omics differential expression analysis of thyroid cancer subtypes. (A) Volcano plot comparing protein expression profiles between papillary thyroid cancer (PTC) and matched adjacent normal tissues (n=37 pairs). Analysis identified 1,864 significantly altered proteins (adjusted p-value < 0.05), with red dots indicating up-regulated and blue dots showing down-regulated proteins in PTC. (B) Transcriptome-wide differential expression analysis between PTC and normal tissues, revealing 1,674 significantly changed genes (adjusted p-value < 0.05). (C) Phosphoproteomic profiling of PTC versus normal tissues, identifying 391 differentially phosphorylated proteins (adjusted p-value < 0.05). (D) Proteomic comparison between pediatric malignant (PM, n=15) and pediatric benign (PB, n=12) thyroid tissues, showing 243 significantly altered proteins (adjusted p-value < 0.05). In all panels, the x-axis represents log2 fold change (FC), and the y-axis shows -log10(adjusted p-value FDR). Red dots indicate significantly up-regulated molecules, while blue dots represent significantly down-regulated molecules. Dashed horizontal lines indicate the significance threshold (adjusted p-value < 0.05).

To comprehensively characterize the molecular landscape of thyroid cancer across different biological levels and age groups, we performed an integrated multi-omics analysis comparing tumor and normal tissues. Figure 2 presents the results of four distinct analytical approaches, each revealing unique aspects of thyroid cancer biology. As shown in Figure 2A, our proteomics comparison between adult PTC tumor and adjacent normal tissues identified 1,864 differentially expressed proteins (adjusted P < 0.05). Among the upregulated proteins, we observed significant elevation of extracellular matrix proteins, including FN1 (Fibronectin 1) and THBS1 (Thrombospondin 1), which are established mediators of tumor invasion and metastasis 16. Conversely, downregulation of HBA1 (Hemoglobin Subunit Alpha 1) and CA2 (Carbonic Anhydrase 2) suggests substantial metabolic reprogramming in tumor tissues 17, 18.

RNA sequencing analysis revealed 1,674 differentially expressed genes, providing insights into transcriptional regulation in PTC (Figure 2B). Notable upregulated genes include IGF2R (Insulin-like Growth Factor 2 Receptor), a key regulator of cell proliferation associated with PTC 19. The downregulation of SLC4A1 (Solute Carrier Family 4 Member 1) indicates significant alterations in cellular ion transport mechanisms 20.

To understand post-translational modifications in PTC, we performed phosphoproteomic analysis in Figure 2C, identifying 391 significantly altered phosphorylation sites. Of particular interest was the altered phosphorylation of TNC (Tenascin C), which plays a crucial role in tumor microenvironment modulation and metastatic progression 21. These findings highlight the importance of protein phosphorylation in cancer development.

To identify age-specific molecular signatures, we compared protein expression between pediatric malignant and benign thyroid tissues (Figure 2D), revealing 243 differentially expressed proteins. Key upregulated proteins included LGALS3 (Galectin 3), an established marker of thyroid malignancy 22, and POSTN (Periostin), which promotes tumor metastasis and angiogenesis 23. This multi-dimensional analysis reveals the complex interplay between transcriptional regulation, protein expression, and post-translational modifications in thyroid cancer. By examining both adult and pediatric cases, our study provides a comprehensive view of the molecular alterations driving thyroid cancer progression across age groups. These findings not only enhance our understanding of thyroid cancer biology but also identify potential therapeutic targets and biomarkers for further investigation.

### The 46 common genes related to cancer reprogramming energy metabolism

**Figure 3:**
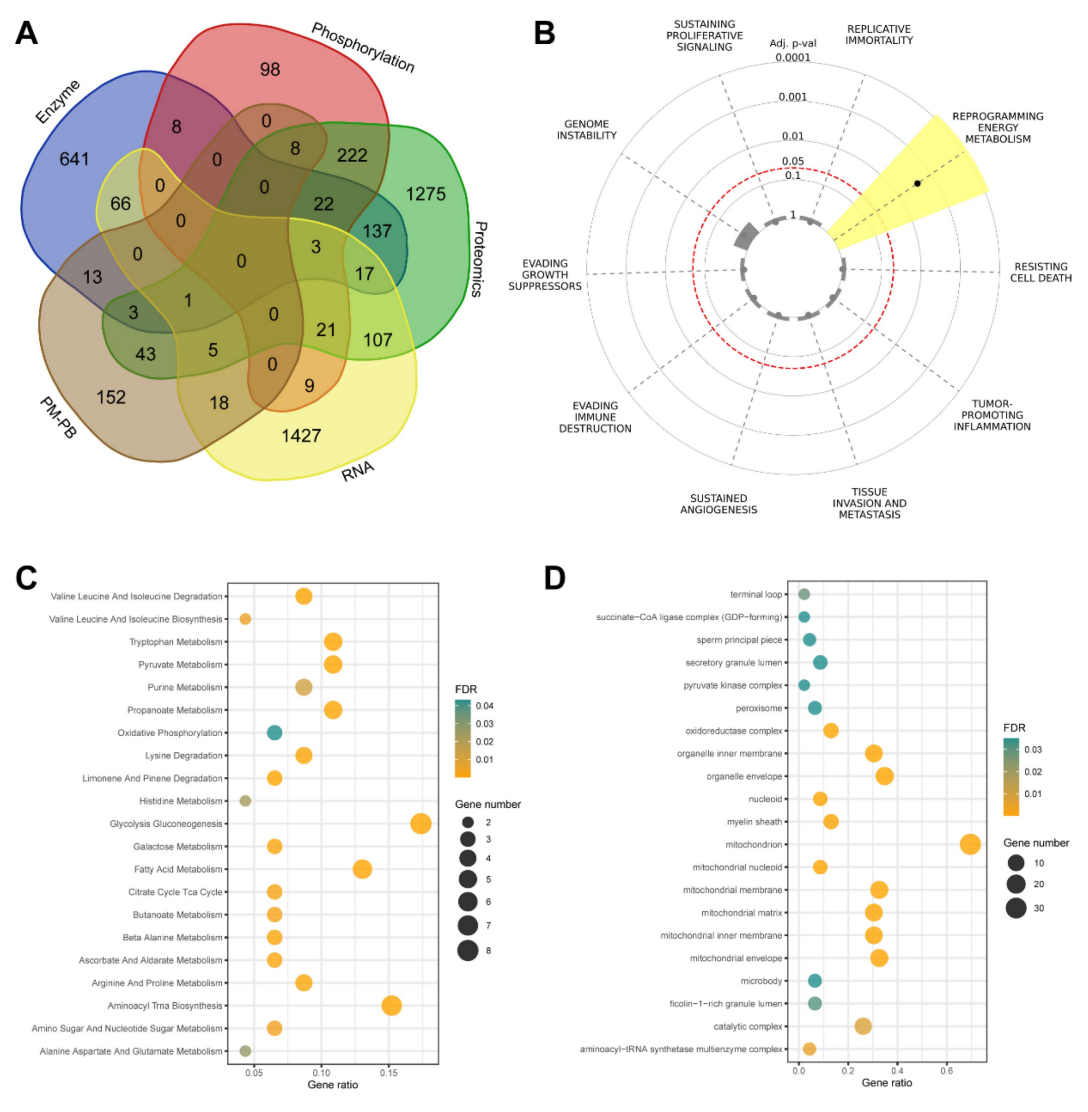
Integration of multi-omics data and functional characterization of overlapping genes in thyroid cancer. (A) Venn diagram illustrating the overlap among five gene/protein lists derived from multi-omics analyses: enzyme, phosphorylation, proteomics, RNA, and pediatric malignant versus pediatric benign (PM/PB) comparisons. The analysis includes data from papillary thyroid cancer (PTC) patients (n=37 pairs of tumor and adjacent normal tissues) and pediatric thyroid tissue samples (PM, n=15; PB, n=12). A total of 46 genes were identified as shared across at least three datasets. (B) Cancer Hallmark analysis of the 46 overlapping genes, highlighting significant enrichment in pathways related to metabolic reprogramming. The radial plot displays adjusted p-values for each hallmark category, with lower values indicating stronger enrichment. (C) Bubble plot showing Gene Ontology (GO) cellular component enrichment among the intersecting genes. Bubble size corresponds to the number of genes in each GO term, and color intensity reflects statistical significance (FDR-adjusted p-value). (D) Bubble plot of enriched KEGG pathways for the overlapping genes. Bubble size represents the gene ratio (proportion of genes in each pathway), and color intensity indicates statistical significance (FDR-adjusted p-value, shown as -log10 values).

To leverage the existing metabolomics data, we utilized the MetaBridge tool to map 334 differentially expressed metabolites to their corresponding KEGG (Kyoto Encyclopedia of Genes and Genomes) enzymes 9. In this way, we have five gene lists, four from the Figure 2, which have the differentially expressed genes in multi-OMICs data. And the last one mapped enzyme from the metabolites. To understand the metabolic reprogramming in thyroid cancer and identify key metabolic regulators, we performed a set analysis and identified a total of the 46 common genes identified across multiple omics platforms. These genes showed significant enrichment in energy metabolism-related pathways, prompting a detailed investigation of their functional roles and regulatory networks (Figure 3).

Our initial overlap analysis (Figure 3A) revealed 46 genes present in at least three of the five multi-omics datasets. Key metabolic regulators such as IDH1, PGAM1, NDUFS3, and LDHB were identified among these common genes, suggesting their central role in thyroid cancer metabolism. This cross-platform consistency strengthens the evidence for their involvement in disease progression. To contextualize these findings within cancer biology, we performed Cancer Hallmark Annotation analysis (Figure 3B). Several genes, including IDH1, PGAM1, SUCLG2, and LDHB, showed significant association with "Reprogramming Energy Metabolism." This enrichment reflects the fundamental metabolic alterations in cancer cells, particularly the Warburg effect and TCA cycle modifications. Notably, IDH1 mutations are established cancer drivers through oncometabolite production, while PGAM1 enhances cancer cell survival by regulating glycolysis and biosynthesis pathways.

To determine the subcellular localization of these metabolic regulators, we performed Gene Ontology cellular component analysis (Figure 3C). The results revealed significant enrichment in mitochondrial compartments, particularly the inner membrane and matrix, highlighting the importance of oxidative phosphorylation and TCA cycle regulation. Additionally, enrichment in catalytic and oxidoreductase complexes emphasizes their crucial role in cellular redox balance and energy production. Further pathway analysis using KEGG (Figure 3D) identified significant enrichment in key metabolic processes. These included glycolysis/gluconeogenesis, citrate cycle, oxidative phosphorylation, and purine metabolism pathways. The visualization of these enrichments through bubble plots, where size indicates gene count and color represents statistical significance, clearly demonstrates the central role of these pathways in supporting tumor growth and adaptation.

Collectively, our multi-dimensional analysis provides compelling evidence for the fundamental role of metabolic reprogramming in thyroid cancer development. The identification of these 46 genes and their associated pathways not only enhances our understanding of thyroid cancer metabolism but also suggests potential therapeutic targets for future investigation. These findings provide a strong foundation for developing metabolism-targeted therapeutic strategies in thyroid cancer treatment.

### Mutational and Clinical Analysis of Public Genomics Data

To investigate the mutational landscape and clinical implications of 46 key genes using publicly available genomic data, we combined the PTC dataset (500 samples) with the two ATC datasets (190 + 117 samples) and conducted survival analyses across 751 patients. By integrating these mutations with survival outcomes, we aimed to identify biomarkers with potential prognostic and therapeutic relevance.

The mutation frequencies and types in the 46 genes are provided in Figure S1 and Table S2, Mutation types include single-nucleotide variants (MUT), amplifications (AMP), homozygous deletions (HOMDEL), and gene fusions (FUSION). The "Percent Samples Altered" column represents the percentage of samples exhibiting any mutation in each gene, while "Num Samples Altered" indicates the absolute number of samples affected. Notably, genes such as B4GALT1, ALDH1A1, NDUFV2, and ACO1 had mutations in 6% of samples, making them frequent mutational targets in thyroid cancer. Among these, NDUFV2 and its closely related family member NDUFS3 highlight the potential involvement of mitochondrial dysfunction in cancer pathophysiology. Similarly, ALDH1A1, alongside other aldehyde dehydrogenase family members such as ALDH4A1 and ALDH3A2, underscores the role of metabolic dysregulation in thyroid cancer progression.

In addition, genes implicated in mitochondrial metabolism, such as GLUD1, SUCLG2, and HADHA, demonstrated alterations in 4%-5% of samples. This observation supports the broader theme of metabolic reprogramming in thyroid cancer, particularly in energy production and oxidative stress responses. The consistent mutation patterns across these genes suggest their potential as candidates for therapeutic intervention and biomarkers for disease stratification.

To provide a comprehensive perspective, we conducted survival analyses across 751 patients (Figure S2). For overall survival (OS), 93 patients were classified into the altered group and 658 into the unaltered group. The median OS for the altered group was significantly shorter at 5.59 months (95% CI: 4.11-9.33), compared to an undefined median OS for the unaltered group due to prolonged survival (p < 0.0001, q < 0.0001). For disease-free survival (DFS), 355 patients were analyzed, with 4 patients in the altered group and 351 in the unaltered group. The altered group again showed a trend toward reduced DFS, although the median survival could not be calculated due to limited events (p = 0.0345, q = 0.0345). These results reinforce the consistent association between mutations in the 46 genes and adverse clinical outcomes across thyroid cancer subtypes.

**Figure 4:**
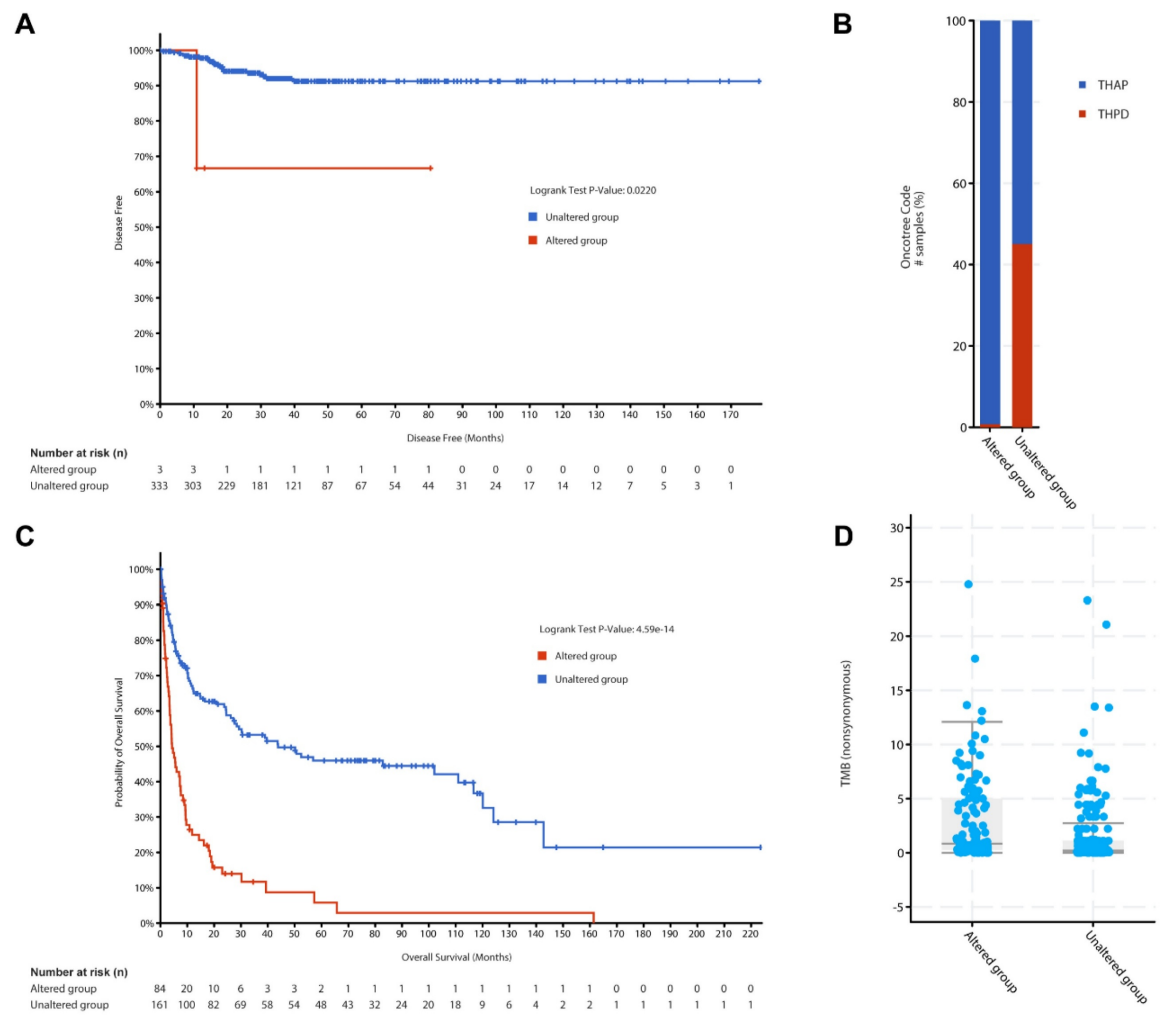
Clinical outcome and molecular characterization of PTC and ATC cohorts of the 46 genes. (A) Disease-free survival analysis for PTC patients stratified by gene alteration status, presented as Kaplan-Meier curves. (B) Proportional representation of altered and unaltered cases across ATC subtypes using Oncotreemap visualization. (C) Overall survival comparison between altered and unaltered ATC patient groups using Kaplan-Meier analysis. (D) Comparative analysis of tumor mutation burden between altered and unaltered ATC groups, displayed as box plots.

To explore the PTC specific feature, Kaplan-Meier survival analysis was performed using data from 336 PTC patients included in the TCGA pan-cancer dataset as shown in Figure 4A. Among these patients, 3 were in the altered group, defined by mutations in the 46 genes, and 333 were in the unaltered group. The median disease-free survival (DFS) for both groups could not be calculated due to insufficient events in the altered group. However, the altered group demonstrated significantly reduced DFS compared to the unaltered group (p = 0.022, q = 0.0439). Additionally, an overall survival analysis of 478 PTC samples (8 in the altered group and 470 in the unaltered group) showed no significant difference between the groups (p = 0.692). These findings suggest that while mutations in the 46 genes are associated with recurrence risks in PTC, they may not significantly affect overall survival outcomes. This dataset underscores the importance of these genetic alterations in influencing early disease recurrence while highlighting the need for further studies to assess their impact on long-term survival.

Building on these findings, we extended the analysis to ATC, an aggressive subtype of thyroid cancer, to explore whether these genes exhibit a similar prognostic significance. The ATC cohort comprised data from two datasets: (1) 190 samples analyzed by whole-genome or whole-exome sequencing, representing ATC and co-occurring differentiated thyroid carcinoma (DTC), and (2) 117 samples subjected to targeted sequencing of 341 cancer genes from patient-derived poorly differentiated thyroid carcinoma (PDTC) and ATC samples.

More interesting, Figure 4B classified ATC samples based on the presence or absence of mutations in the 46 genes. The x-axis represents oncotrack classifications, while the y-axis indicates the proportion of samples within each group. A substantial proportion of ATC samples were categorized as altered, demonstrating a strong correlation between mutations in these genes and aggressive ATC subtypes. This classification underscores the heterogeneity within ATC and highlights the potential utility of these genes in refining molecular subtypes and predicting clinical behavior.

To further explore the clinical implications of these mutations in ATC, we analyzed overall survival (Figure 4C) and tumor mutation burden (TMB, Figure 4D), two critical indicators of disease aggressiveness and therapeutic potential. Survival analysis revealed stark differences between the altered and unaltered groups. The x-axis denotes OS time (in months), and the y-axis represents survival probability. The altered group exhibited a dramatically shorter OS compared to the unaltered group (log-rank test, p = 4.59 × 10^-14^). Among the 84 patients in the altered group, 69 events (deaths) occurred, with a median overall survival of 4.34 months (95% CI: 3.52-7.62). Conversely, the unaltered group, consisting of 161 patients with 83 events, exhibited a significantly longer median overall survival of 43.78 months (95% CI: 27.17-116.68). These results underscore the potential of these mutations as prognostic markers, as their presence is strongly associated with poorer outcomes and more aggressive disease phenotypes in ATC. This finding is consistent with the hypothesis that mutations in the 46 genes play a critical role in ATC progression. The findings from all the survival analyses highlight the clinical relevance of the 46-gene panel in thyroid cancer. Specifically, these genetic alterations are associated with poorer survival outcomes and increased recurrence risks in both PTC and ATC, with a more pronounced impact observed in ATC. By integrating genomic and clinical data, this study provides a robust foundation for further exploration of these mutations as potential biomarkers or therapeutic targets in thyroid cancer.

A box plot analysis compared the TMB in Figure 4D, defined as the number of nonsynonymous mutations per megabase, between the altered and unaltered groups in the ATC cohort. The x-axis shows the group classification, while the y-axis displays TMB scores. The altered group demonstrated significantly higher TMB, a feature often linked to poor prognosis and increased immunogenicity in cancer. These findings suggest that mutations in the 46 genes contribute to the mutational landscape of ATC, emphasizing their potential as biomarkers for immunotherapy responsiveness and personalized treatment strategies.

Collectively, these findings provide a comprehensive understanding of the mutational landscape of the 46 genes in thyroid cancer. By integrating clinical and genomic data, this study highlights the importance of these genes in shaping disease outcomes, offering promising avenues for further research and clinical application.

### Immunotherapy Analysis of Key Genes in Thyroid Cancer

To assess the potential application of 46 clinically significant genes in immunotherapy for thyroid cancer, we analyzed their expression in a publicly available anti-PD1 gene expression dataset (Table S3). The analysis revealed a robust stratification of the 46 genes based on their statistical significance in relation to immunotherapy response. Genes with the seven most significant p-values (<1.0E-04) and false discovery rates (FDR ≤ 1%) include AK2, SUCLG2, NDUFV2, GLUD1, HADHA, ALDH1A1, NADSYN1, marking them as the most promising candidates for further exploration. A subset of genes, such as CHKB, GCDH, and OPA1, exhibited moderate significance (FDR = 10%), suggesting their roles may be context dependent. Genes with higher p-values (FDR > 50%), including PPA2, UGDH, and CRAT, may contribute indirectly to immunotherapy response through secondary or complementary pathways.

**Figure 5:**
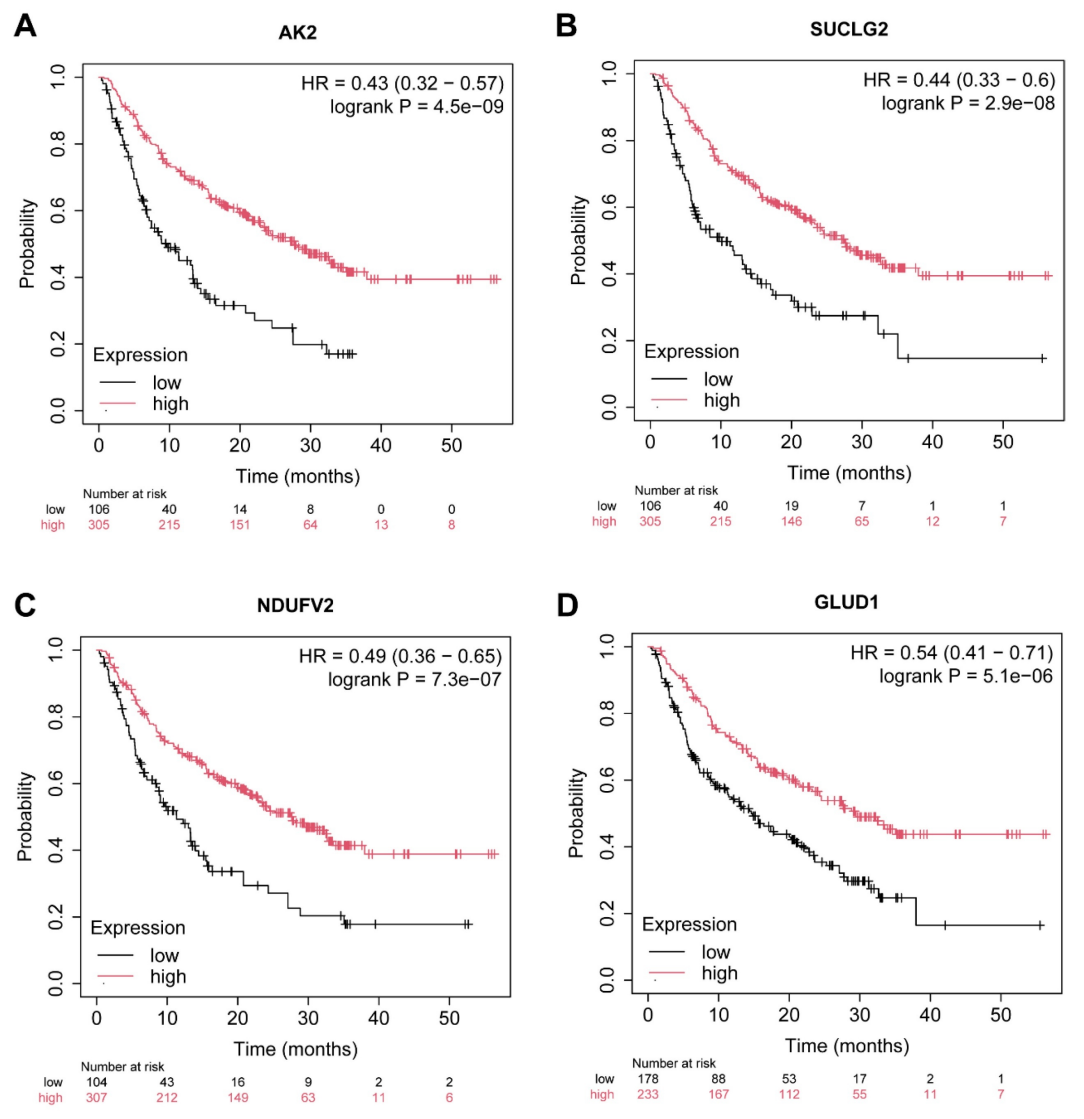
Kaplan-Meier survival analysis of key metabolic gene expression in relation to anti-PD-1 immunotherapy response in thyroid cancer. (A-D) Kaplan-Meier survival curves illustrating the association between expression levels of four metabolic genes—AK2 (A), SUCLG2 (B), NDUFV2 (C), and GLUD1 (D)—and overall survival in patients treated with anti-PD-1 immunotherapy (total n = 520). Patients were stratified into high (red) and low (black) expression groups based on median gene expression values. The x-axis represents time in months, and the y-axis indicates the probability of survival. Hazard ratios (HR) with 95% confidence intervals and log-rank test p-values are displayed in each panel to assess statistical significance. The number of patients at risk at each time point is shown below each plot.

Among these genes, 40 were successfully mapped to the dataset, with 30 demonstrating significant associations with immunotherapy response (P-value < 0.05). The top four genes exhibiting the most significant associations—AK2, SUCLG2, NDUFV2, and GLUD1—are highlighted and described below (Figure 5A-D).

As shown in Figure 5A, AK2 (Adenylate Kinase 2) demonstrated the most significant association with immunotherapy outcomes (HR = 0.43, p = 4.5E-09). As a regulator of cellular energy homeostasis, AK2 plays a critical role in maintaining mitochondrial function, which is essential for immune cell activation 24. Its high expression is strongly correlated with improved immunotherapy response, potentially due to its role in sustaining energy balance under immune stress conditions.

SUCLG2 (Succinate-CoA Ligase GDP-Forming Beta Subunit) was the second most significant gene (Figure 5B, HR = 0.51, p = 2.9E-08). This gene is a key component of the tricarboxylic acid (TCA) cycle and is involved in mitochondrial energy metabolism. High SUCLG2 expression is associated with enhanced immunotherapy response 25, potentially by modulating succinate metabolism, a process implicated in immune cell activation and tumor microenvironment reprogramming.

Interestingly, NDUFV2 (NADH: Ubiquinone Oxidoreductase Core Subunit V2) also exhibited significant association with immunotherapy efficacy (HR = 0.47, p = 7.3E-07) in Figure 5C. As a component of Complex I in the mitochondrial electron transport chain, NDUFV2 contributes to energy production and redox balance 26. This function is critical for immune cell metabolism and tumor immune surveillance. Additionally, the related gene NDUFS3 (HR = 0.55, p = 7.0e-03) underscores the importance of mitochondrial function in modulating the immune response, further supporting the role of the NDUFV gene family in immunotherapy outcomes.

The fourth gene GLUD1 (Glutamate Dehydrogenase 1) was also significantly associated with improved immunotherapy response as depicted in Figure 5D (HR = 0.54, p = 5.1E-06). GLUD1 is integral to glutamine metabolism, a process critical for cancer cell survival and immune cell function 27. Its high expression suggests a dual role in modulating tumor metabolism and enhancing the anti-tumor immune response.

Additional genes showing significant associations include ALDH1A1 (HR = 0.56, p = 9.6e-05) and HADHA (HR = 0.53, p = 1.5e-05), which emphasize the importance of metabolic pathways in immunotherapy response. ALDH1A1 is involved in cellular detoxification and retinoic acid signaling, which may contribute to immune modulation, while HADHA's role in fatty acid β-oxidation influences tumor microenvironment reprogramming. Genes involved in mitochondrial function and energy metabolism, such as NADSYN1, GPD1, and IARS2, also demonstrated significant correlations with immunotherapy outcomes. These genes collectively highlight the centrality of mitochondrial integrity and metabolic adaptability in shaping immune responses.

In sum, this systematic categorization underscores the pivotal role of mitochondrial energy metabolism, glutamine metabolism, and redox homeostasis in the efficacy of anti-PD1 therapies in thyroid cancer.

### Validating the oncogenic role of AK2 in thyroid cancer cell lines

To validate one of the top seven prognostic gene candidates, we selected AK2 to investigate its potential as a biomarker for stratifying thyroid cancer progression and treatment. To this aim, we used CAL-62 and KTC-1 cells, which are well-established human thyroid cancer cell lines commonly used to model anaplastic and papillary thyroid carcinomas, respectively. CAL-62 originates from an undifferentiated thyroid carcinoma and exhibits aggressive, mesenchymal-like features, while KTC-1 derives from papillary carcinoma and retains characteristics of differentiated thyroid cancer.

**Figure 6:**
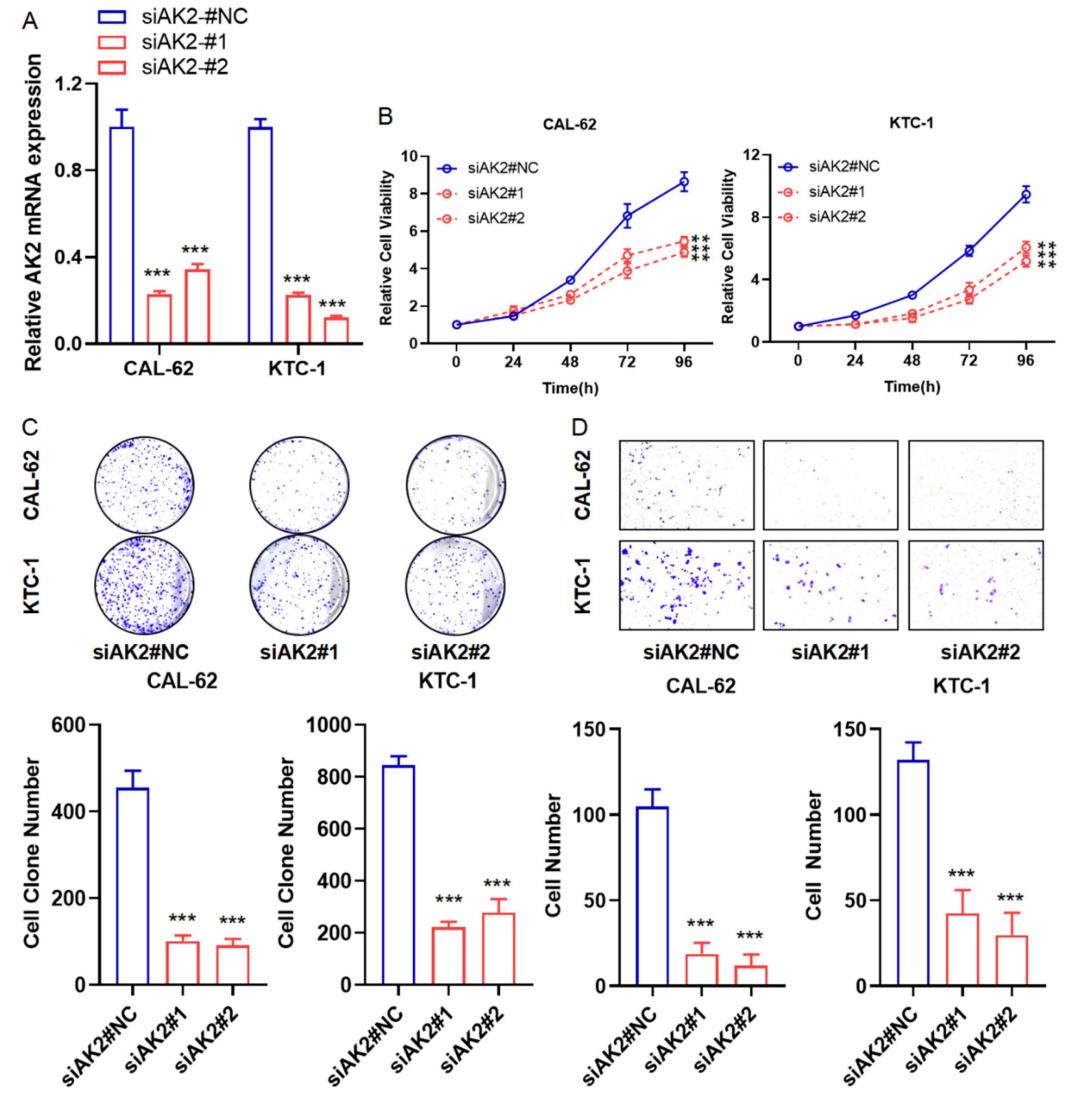
AK2 functions as an oncogene and promotes proliferation and migration in thyroid cancer cell lines. (A) Quantitative PCR analysis of AK2 mRNA expression in CAL-62 (anaplastic thyroid cancer) and KTC-1 (papillary thyroid cancer) cells following transfection with AK2-targeting siRNA, confirming effective knockdown. (B) Cell proliferation was assessed using the CCK-8 assay in AK2-knockdown CAL-62 and KTC-1 cells, showing reduced viability compared to controls. (C) Colony formation assay demonstrated a significant decrease in the long-term proliferative capacity of both cell lines after AK2 knockdown. (D) Transwell migration assay revealed that suppression of AK2 markedly inhibited the migratory ability of CAL-62 and KTC-1 cells, with near-complete loss of migration observed in CAL-62.

As shown in Figure 6A, AK2 mRNA expression was significantly reduced in CAL-62 and KTC-1 cells following siRNA-mediated knockdown. Cell viability assays revealed that AK2 knockdown significantly inhibited the proliferation of both cell lines (Figure 6B). Consistently, colony formation assays showed a marked reduction in colony-forming ability (Figure 6C). Transwell migration assays further demonstrated that AK2 silencing substantially impaired cell migration, with CAL-62 cells exhibiting near-complete loss of migratory capacity (Figure 6D). These findings suggest that AK2 plays a critical role in thyroid cancer cell proliferation and migration, supporting its potential as a biomarker and therapeutic target for personalized treatment strategies.

## Discussion

Our study aimed to identify conserved pathways and genes across multiple omics platforms in both adult and pediatric thyroid cancer cohorts, with a focus on integrating proteomics, phosphoproteomics, metabolomics, and transcriptomics datasets. By employing a comprehensive cross-platform analysis, we identified 46 genes consistently altered across multiple molecular dimensions. These genes were predominantly associated with mitochondrial energy metabolism 28, underscoring the significance of metabolic adaptations in thyroid cancer progression. This multi-layered approach provided robust validation of these molecular signatures across diverse experimental conditions and patient populations.

A significant strength of our study lies in its comparative analysis of adult and pediatric PTC datasets, revealing shared molecular mechanisms between these age-distinct presentations of thyroid cancer 3. Our integrative analysis of the 46 genes identified across multiple omics layers revealed notable correlations between changes in protein expression and other molecular features, which reveal their functional roles in thyroid cancer biology. For example, several genes such as IDH1, PGAM1, and LDHB demonstrated consistent upregulation at both the transcript and protein levels, underscoring their enhanced activity in metabolic reprogramming pathways like glycolysis and the TCA cycle. These coordinated changes suggest that alterations at the mRNA level translate into increased protein abundance, potentially amplifying their effects on energy production and redox balance within tumor cells. Moreover, phosphorylation state modifications of proteins like TNC, which is involved in metastasis and microenvironment modulation, further indicate post-translational regulation that could influence tumor progression and metastatic potential.

Given the complexity inherent in multi-OMICs data integration, our study employed several strategies to mitigate potential confounders such as batch effects, technical variability, tumor purity, and cellular heterogeneity. Rigorous normalization procedures and statistical corrections, including false discovery rate adjustments, were applied across datasets to reduce technical biases and improve comparability between different omics layers, such as proteomics, phosphoproteomics, metabolomics, and transcriptomics. Additionally, enzyme mapping of metabolites using tools like MetaBridge helped standardize pathway-level interpretations, further minimizing confounding due to disparate data sources. Where available, clinical metadata such as tumor cellularity estimates were incorporated to account for heterogeneity, and pathway enrichment analyses helped focus on biological processes likely to be genuine rather than artifacts of sample variation. These methodologies collectively enhanced the robustness of the integrated analyses, although residual confounding cannot be eliminated, underscoring the importance of continued refinement with prospective, carefully controlled datasets.

Furthermore, the observed multi-OMICs relationships, especially those involving key genes implicated in energy metabolism, suggest that the coordinated changes across different molecular layers are likely reflective of true biological mechanisms rather than technical artifacts. For instance, several genes such as IDH1 and PGAM1 displayed consistent alterations at the transcript, protein, and metabolite levels, indicating their critical role in reprogramming energy pathways like glycolysis and the TCA cycle in thyroid cancer. Changes in phosphorylation states of proteins such as TNC further implicate post-translational modifications in functional regulation during tumor progression. These multi-OMICs relationships highlight the interconnected nature of gene expression, enzyme activity, and metabolite dynamics, which collectively influence tumor growth, immune evasion, and response to therapy. The functional consequences of these integrated molecular alterations underscore potential vulnerabilities that can be targeted therapeutically, for example, through drugs that inhibit key metabolic enzymes or modulate post-translational modifications, thereby disrupting cancer cell metabolism and microenvironment interactions.

Reflecting on this investigation, several limitations warrant consideration. The reliance on a specific cohort may restrict the generalizability of the findings, as biological variability can differ widely in broader populations. One notable limitation is the disparity in cohort sizes between adult and pediatric thyroid cancer samples, as well as between different subtypes such as PTC and ATC. Although our integrated multi-omics approach allowed for robust identification of key molecular signatures, the smaller size of certain cohorts, particularly pediatric samples, may limit the statistical power and the ability to detect less prominent alterations. Larger, more diverse cohorts would help validate and extend these findings, ensuring the generalizability across different patient populations. Furthermore, while the integrative multi-omics approach provides a comprehensive dataset, the complexity of data interpretation poses challenges, potentially overlooking significant interactions among molecular pathways 29.

The analysis of anti-PD-1 immunotherapy response in 520 patients further highlighted several genes significantly associated with treatment efficacy, particularly those involved in metabolic processes. Notable genes, including AK2, SUCLG2, NDUFV2, GLUD1, and HADHA, emerged as potential biomarkers for predicting immunotherapy response. These findings suggest actionable targets for enhancing immunotherapeutic strategies and overcoming treatment resistance in aggressive thyroid cancers 30.

The association of the 46-gene panel with poorer survival outcomes reinforces their potential as prognostic markers, aiding in risk stratification and personalized treatment approaches. By bridging molecular research with clinical practice, our study not only provides a foundation for precision medicine but also highlights actionable pathways for therapeutic intervention. Ultimately, the integrative multi-omics approach presented here offers valuable insights into thyroid cancer biology, paving the way for the development of targeted therapies and personalized treatment strategies aimed at improving patient outcomes.

## Conclusions

This integrative multi-omics study significantly advances our understanding of thyroid cancer by uncovering critical metabolic pathways and identifying prognostic biomarkers. The consistent alterations in key genes across proteomics, phosphoproteomics, metabolomics, and transcriptomic data underscore the importance of energy metabolism reprogramming in thyroid cancer progression. Moreover, the associations between specific gene mutations and clinical outcomes, including survival and immunotherapy response, highlight the potential for these genes to serve as actionable targets in personalized medicine. Future research should focus on experimental validation of these findings and the development of targeted therapies to improve patient outcomes in thyroid cancer.

## Supplementary Material

Supplementary figures and table legends.

Supplementary table 1.

Supplementary table 2.

Supplementary table 3.

## Abbreviations

PTC: papillary thyroid carcinoma
ATC: anaplastic thyroid carcinoma
TCGA: The Cancer Genome Atlas
RNAseq: RNA sequencing
DEG: Differentially expressed gene
GEO: Gene Expression Omnibus
GO: Gene Ontology
KEGG: Kyoto Encyclopedia of Genes and Genomes

## References

[1] Cabanillas ME, McFadden DG, Durante C (2016). Thyroid cancer. Lancet.

[2] Vanden Borre P, Schrock AB, Anderson PM, Morris JC 3rd, Heilmann AM, Holmes O (2017). Pediatric, Adolescent, and Young Adult Thyroid Carcinoma Harbors Frequent and Diverse Targetable Genomic Alterations, Including Kinase Fusions. Oncologist.

[3] Wang Z, Wang H, Zhou Y, Li L, Lyu M, Wu C (2024). An individualized protein-based prognostic model to stratify pediatric patients with papillary thyroid carcinoma. Nat Commun.

[4] Zhu H, Li Y, Guo J, Feng S, Ge H, Gu C (2023). Integrated proteomic and phosphoproteomic analysis for characterization of colorectal cancer. J Proteomics.

[5] Qu N, Chen D, Ma B, Zhang L, Wang Q, Wang Y (2024). Integrated proteogenomic and metabolomic characterization of papillary thyroid cancer with different recurrence risks. Nat Commun.

[6] Schmidt DR, Patel R, Kirsch DG, Lewis CA, Vander Heiden MG, Locasale JW (2021). Metabolomics in cancer research and emerging applications in clinical oncology. CA Cancer J Clin.

[7] Buzdin A, Sorokin M, Garazha A, Glusker A, Aleshin A, Poddubskaya E (2020). RNA sequencing for research and diagnostics in clinical oncology. Semin Cancer Biol.

[8] Heo YJ, Hwa C, Lee GH, Park JM, An JY (2021). Integrative Multi-Omics Approaches in Cancer Research: From Biological Networks to Clinical Subtypes. Mol Cells.

[9] Blimkie T, Lee AH, Hancock REW (2020). MetaBridge: An Integrative Multi-Omics Tool for Metabolite-Enzyme Mapping. Curr Protoc Bioinformatics.

[10] Love MI, Huber W, Anders S (2014). Moderated estimation of fold change and dispersion for RNA-seq data with DESeq2. Genome Biol.

[11] Lam F, Lalansingh CM, Babaran HE, Wang Z, Prokopec SD, Fox NS (2016). VennDiagramWeb: a web application for the generation of highly customizable Venn and Euler diagrams. BMC Bioinformatics.

[12] Menyhart O, Kothalawala WJ, Győrffy B A gene set enrichment analysis for the cancer hallmarks. Journal of Pharmaceutical Analysis. 2024: 101065.

[13] Chen J, Bardes EE, Aronow BJ, Jegga AG (2009). ToppGene Suite for gene list enrichment analysis and candidate gene prioritization. Nucleic Acids Res.

[14] Gao J, Aksoy BA, Dogrusoz U, Dresdner G, Gross B, Sumer SO (2013). Integrative analysis of complex cancer genomics and clinical profiles using the cBioPortal. Sci Signal.

[15] Kovacs SA, Fekete JT, Gyorffy B (2023). Predictive biomarkers of immunotherapy response with pharmacological applications in solid tumors. Acta Pharmacol Sin.

[16] Hu C, Wen J, Gong L, Chen X, Wang J, Hu F (2017). Thrombospondin-1 promotes cell migration, invasion and lung metastasis of osteosarcoma through FAK dependent pathway. Oncotarget.

[17] Hope C, Robertshaw A, Cheung KL, Idris I, English E (2016). Relationship between HbA1c and cancer in people with or without diabetes: a systematic review. Diabet Med.

[18] Nogradi A (1998). The role of carbonic anhydrases in tumors. Am J Pathol.

[19] Vella V, Sciacca L, Pandini G, Mineo R, Squatrito S, Vigneri R (2001). The IGF system in thyroid cancer: new concepts. Mol Pathol.

[20] Gorbatenko A, Olesen CW, Boedtkjer E, Pedersen SF (2014). Regulation and roles of bicarbonate transporters in cancer. Front Physiol.

[21] Nagaharu K, Zhang X, Yoshida T, Katoh D, Hanamura N, Kozuka Y (2011). Tenascin C induces epithelial-mesenchymal transition-like change accompanied by SRC activation and focal adhesion kinase phosphorylation in human breast cancer cells. Am J Pathol.

[22] Chiu CG, Strugnell SS, Griffith OL, Jones SJ, Gown AM, Walker B (2010). Diagnostic utility of galectin-3 in thyroid cancer. Am J Pathol.

[23] Zhu M, Fejzo MS, Anderson L, Dering J, Ginther C, Ramos L (2010). Periostin promotes ovarian cancer angiogenesis and metastasis. Gynecol Oncol.

[24] Chou J, Alazami AM, Jaber F, Hoyos-Bachiloglu R, Jones J, Weeks S (2020). Hypomorphic variants in AK2 reveal the contribution of mitochondrial function to B-cell activation. J Allergy Clin Immunol.

[25] Zhang X, Liu J, Cheng Y, Chen K, Chen Y, Zhu H (2023). Metabolic enzyme Suclg2 maintains tolerogenicity of regulatory dendritic cells diffDCs by suppressing Lactb succinylation. J Autoimmun.

[26] Oruganty-Das A, Ng T, Udagawa T, Goh EL, Richter JD (2012). Translational control of mitochondrial energy production mediates neuron morphogenesis. Cell Metab.

[27] Wang L, Fang Z, Gao P, Zheng J (2022). GLUD1 suppresses renal tumorigenesis and development via inhibiting PI3K/Akt/mTOR pathway. Front Oncol.

[28] Wallace DC (2012). Mitochondria and cancer. Nat Rev Cancer.

[29] Hernandez-Lemus E, Ochoa S (2024). Methods for multi-omic data integration in cancer research. Front Genet.

[30] Pinheiro Neto A, Lucchesi HL, Valsecchi V, Ward LS, Cunha LL (2024). Immunotherapy for patients with thyroid cancer: a comprehensive appraisal. Chin Clin Oncol.

