# Supplementary material for: Integrative Multi-Omics Analysis Reveals Key Metabolic Regulators and Prognostic Biomarkers in Pediatric and Adult Thyroid Cancer
Source: J Cancer. 2025 Jul 28;16(11):3551–66. doi: 10.7150/jca.117034 (PMC12374938; doi:10.7150/jca.117034)
Supplement: Supplementary file 1 — Supplementary figures and table legends. [file jcav16p3551s1.pdf]

Supplementary files

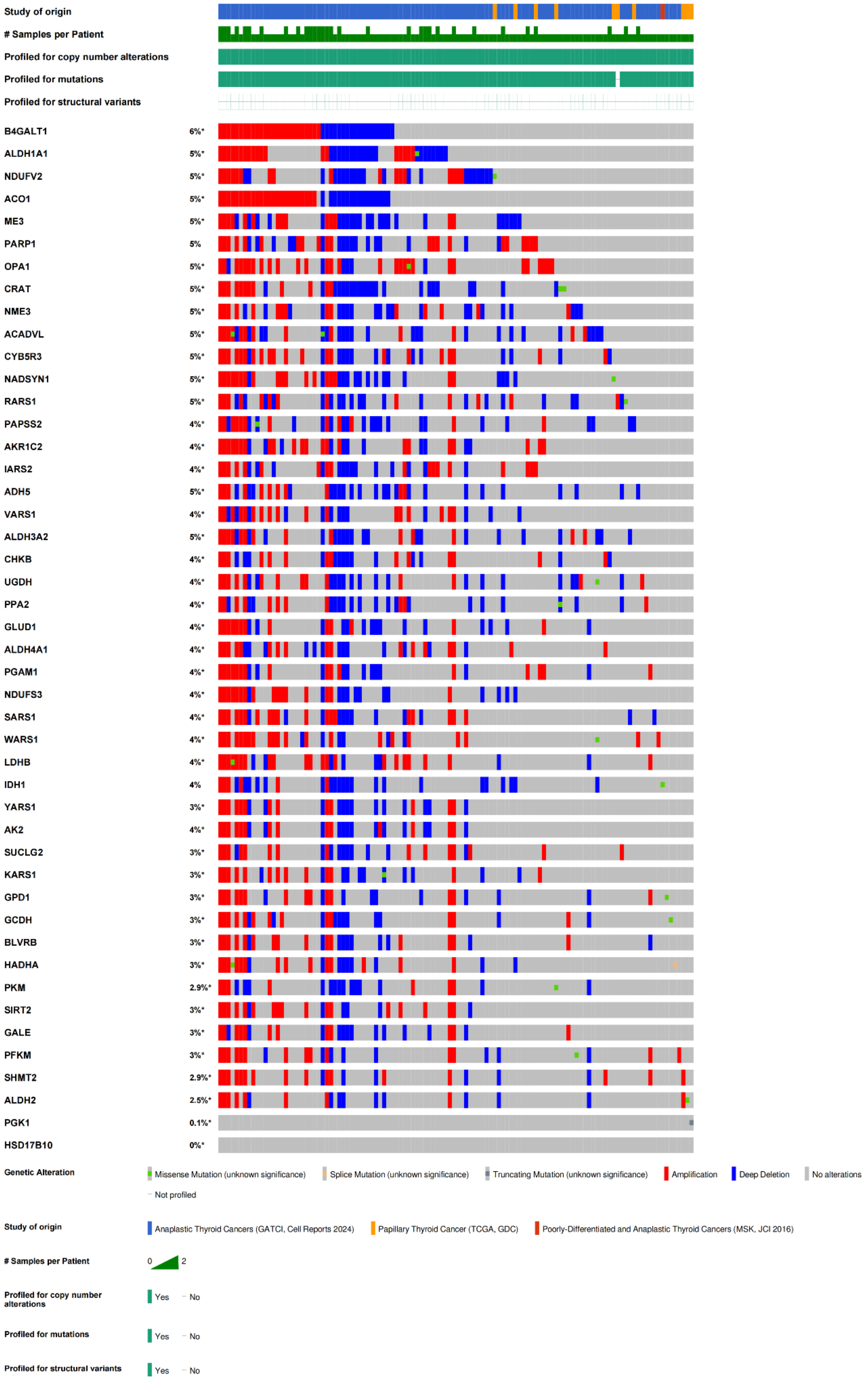

Figure S1. OncoPrint visualization of genomic alterations in 46 key genes across papillary thyroid cancer (PTC) and anaplastic thyroid cancer (ATC) cohorts. OncoPrint plot summarizing the landscape of genomic alterations—including copy number alterations, mutations, and structural variants—in 46 key genes across three combined thyroid cancer cohorts: papillary thyroid cancer (PTC) and anaplastic thyroid cancer (ATC). The analysis includes a total of 751 patients. Each column represents an individual patient, and each row corresponds to a gene. Red indicates copy number gains/amplifications; Blue indicates copy number losses/deletions; Green indicates mutations; Gray indicates no detected alteration. The top bars summarize the number and type of alterations per patient.

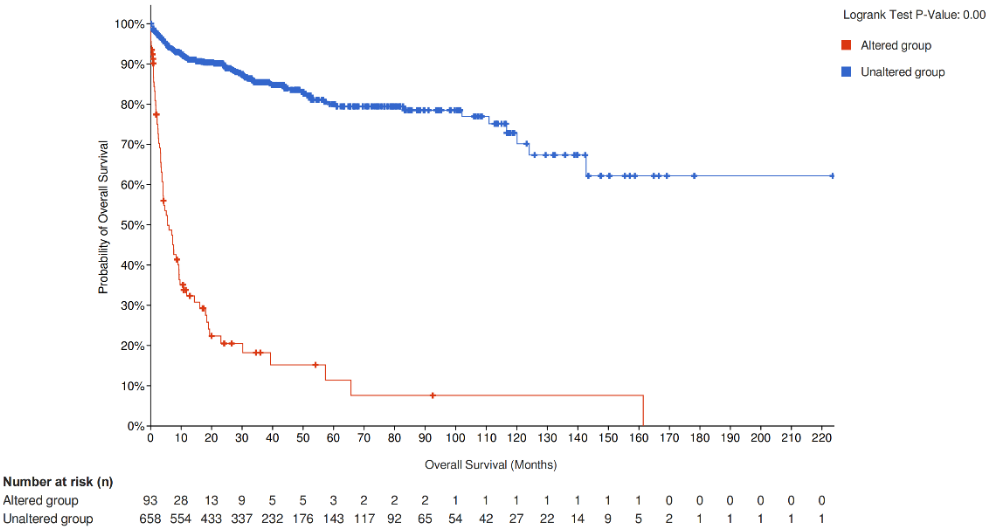

Figure S2. Kaplan-Meier overall survival analysis based on genomic alterations in 46 key genes across combined papillary thyroid cancer (PTC) and anaplastic thyroid cancer (ATC) cohorts. Kaplan-Meier survival curves comparing overall survival between patients with (red, "Altered group", n=93) and without (blue, "Unaltered group", n=658) genomic alterations in any of the 46 key metabolic genes. The analysis includes a total of 751 patients from three combined cohorts of PTC and ATC (see Methods for cohort details and sample breakdown). The x-axis represents overall survival time in months, and the y-axis indicates the probability of overall survival. The number of patients at risk at each time point is shown below the plot.
Statistical significance of survival differences between groups was assessed using the log-rank test (p-value shown on the plot).

Table S1: List of 46 genes with consistent changes in multi-OMICs data integration.
Table S2: Pan-cancer mutation rates of the 46 genes.
Table S3: Anti-PD1 immunotherapy analysis of the 46 genes.
